# The Whole Truth

**Published:** 1910-09-24

**Authors:** 


### The Whole Truth.

In a certain Section of the recent British Medical Association Annual Meeting the following little scene took place. The author of a certain paper (after, it is true, apologising for the abstract way in which he meant to treat the subject under discussion by the Section) proceeded to read out rapidly, in a monotonous tone of voice, stuff which had remarkedly small relevance, and not much coherence even. After a while the audience relinquished its forward bent of attention. Members straightened up, threw back their heads, then began to look vexed—before long coughed, shuffled with their feet, and initiated a crescendo chorus of interjection. Still the reader hurried on; until the chairman, who manifestly thought as the majority did, and who later on in a short speech gave evidence of excellent critical ability, got up and warned him he was wide of the point. He replied, half airily, half nervously, that he had nearly done; but as matters did not improve, and the patience of the Section seemed exhausted, the chairman rose again shortly and stopped him. The speaker returned to his seat with his paper unfinished.

Now of this sectional meeting three accounts have since appeared in the medical papers. One of them described the contribution in question as a valuable paper; none gave the faintest hint of the lack of appetite shown by the audience for this particular item on the mental bill of fare, or of the little display of discipline above mentioned. Some pain was thereby saved to one person, perhaps also to those responsible for the selection of the paper; also space was economised: but the question is, Was this composite gain worth the sacrifice of candour it cost?

It was the late George Meredith, we believe, who pointed out the soapiness of present-day literary criticism. So if a writer of this calibre thought it worth while to deplore publicly the lack of rigour shown in dealing with Tom, Dick, and Harry as authors, it is allowable for us not to be deterred from comment by the intrinsic unimportance of the above incident. The suppression of painful facts is a good thing one can very easily indeed have too much of. That is the point, not at all the standing of the person who benefits thereby. In this case it happens not to have been a leader of the profession; but it would be easy to give instances where the pen is "dipped in butter" to serve someone who is.

Take, for example, the publications—manuals, special journals, and so forth—which one and all make a "highly successful first appearance," and are carried on under the "able editorship" of Dr. So-and-So; whereas the publishers are distributing free copies and making desperate efforts to obtain a circulation, while serious students of the subject go to Continental sources for information. Of the many evil consequences of such insincerity some will readily occur to the mind, but there is one we may mention which is not at once apparent, but yet very formidable. Everyone comes to take it for granted that everything which is not glaringly bad will receive conventional praise. Accordingly reputation comes to be made not by written comment, which can be well considered and reasoned, but by conversation, even by chance remarks. Hence an opportunity for wire-pulling and caballing, a premium on social adaptability; things which in some measure may have a legitimate place in political and business life, but which happily are beneath the plane of scientific thought. Whenever the voice of the unmistakable crank is heard in the land, or whenever a rather distinguished member of the profession, fancying himself a second Sir Thomas Browne, begins to behave like Swift's Æolists, it is the duty of the intelligent recorder delicately, but faithfully, to indicate the painful fact.

